# Effect of Calcium and Potassium on Antioxidant System of *Vicia faba* L. Under Cadmium Stress

**DOI:** 10.3390/ijms13066604

**Published:** 2012-05-29

**Authors:** Manzer H. Siddiqui, Mohamed H. Al-Whaibi, Ahmed M. Sakran, Mohammed O. Basalah, Hayssam M. Ali

**Affiliations:** Department of Botany and Microbiology, College of Science, King Saud University, Riyadh, Saudi Arabia; E-Mails: mwhaibi@ksu.edu.sa (M.H.A.-W.); ahmed_sakran@yahoo.com (A.M.S.); basalah@ksu.edu.sa (M.O.B.); hayssam77@hotmail.com (H.M.A.)

**Keywords:** antioxidant, faba bean, heavy metal, nutrient content, photosynthetic pigments

## Abstract

Cadmium (Cd) in soil poses a major threat to plant growth and productivity. In the present experiment, we studied the effect of calcium (Ca^2+^) and/or potassium (K^+^) on the antioxidant system, accumulation of proline (Pro), malondialdehyde (MDA), and content of photosynthetic pigments, cadmium (Cd) and nutrients, *i.e.*, Ca^2+^ and K^+^ in leaf of *Vicia faba* L. (cv. TARA) under Cd stress. Plants grown in the presence of Cd exhibited reduced growth traits [root length (RL) plant^−1^, shoot length (SL) plant^−1^, root fresh weight (RFW) plant^−1^, shoot fresh weight (SFW) plant^−1^, root dry weight (RDW) plant^−1^ and shoot dry weight (SDW) plant^−1^] and concentration of Ca^2+^, K^+^, Chlorophyll (Chl) *a* and Chl *b* content, except content of MDA, Cd and (Pro). The antioxidant enzymes [peroxidase (POD) and superoxide dismutase (SOD)] slightly increased as compared to control under Cd stress. However, a significant improvement was observed in all growth traits and content of Ca^2+^, K^+^, Chl *a*, Chl *b*, Pro and activity of antioxidant enzymes catalase (CAT), POD and SOD in plants subjected to Ca^2+^ and/or K^+^. The maximum alleviating effect was recorded in the plants grown in medium containing Ca^2+^ and K^+^ together. This study indicates that the application of Ca^2+^ and/or K^+^ had a significant and synergistic effect on plant growth. Also, application of Ca^2+^ and/or K^+^ was highly effective against the toxicity of Cd by improving activity of antioxidant enzymes and solute that led to the enhanced plant growth of faba bean plants.

## 1. Introduction

Increasing cadmium (Cd) in soil is an enormous constraint for agriculture worldwide. Cd is a highly toxic metal that causes deleterious effects on plants, and limits crop productivity [1]. Cd is commonly released into the arable soil from industries, energy, municipal sources and farming practices, and has been ranked No. 7 among the top 20 toxins [2]. Cd is easily taken up by plant roots and transported to the leaves through xylem [3]. Most plants are sensitive to low concentration of Cd, which disturb the physiological and molecular mechanisms by which plants carry out adaptive response to the environmental stresses. Therefore, it is important to understand the physiological mechanisms by which plants could perform normally under abiotic stress. Cd causes inhibition of physiological process such as photosynthesis, respiration, cell elongation, plant water relationship and assimilation of nitrogen, sulphur and phosphate, resulting in poor growth and development of plants [1,4]. Cd also disturbs the ion homeostasis in plants. The presence of Cd in soil perturbs the absorption of nutrients such as Ca, Mg, K, N, P, Fe, Mn, Cu, Zn and Ni [5–7]. However, the mechanisms involved in its toxicity as well as the cell response against the metal have not been well established [8,9].

Application of nutrients has become indispensable factor that has a broad influence on the growth and metabolism of plants under the condition of different soil environments [10]. One possible approach to minimize the Cd stress on plant productivity is through the addition of mineral nutrients, of which potassium (K^+^) has an outstanding role in plant growth and development. K^+^ is the most abundant cation in plants (up to 10% on dry weight basis) [11]. It is present in high concentration in cytosol and chloroplast, and activates many enzymes by stabilizing the pH between 7 and 8, by changing the enzymatic conformation, and also by binding the enzymes surface [12,13]. A large number of enzymes are either completely dependent on or stimulated by K^+^; more than 50 enzymes are activated by K^+^ [14]. It induces the cell elongation and maintains osmoregulation. K^+^-assimilation is an essential pathway for offsetting the Cd stress in plants by giving stimulatory influence of synthesis of protein, soluble carbohydrates and soluble nitrogen containing compounds (12), hence, these solutes may play a role in osmotic adjustment. Besides K^+^, calcium (Ca^2+^) has a significant role in alleviating the inhibitory effect of Cd on growth and physiological processes [15,16], and also increasing antioxidant enzyme activities, and in reducing lipid peroxidation of cell membranes [16,17]. Ca^2+^ plays an important role in heavy metal detoxification and in tolerance of plant to biotic and abiotic stress [18–20]. Ca^2+^ has also been shown to stabilize cell membrane surfaces, influence the pH of cells and prevent solute leakage from cytoplasm [21]. It is required for various structural roles in the cell wall and membranes; it is a counteraction for inorganic and organic anions in the vacuole, and the cytosolic. Ca^2+^ concentration is one of many cellular network parameters orchestrating complex cellular signaling coordinating responses to numerous developmental cues and environmental challenges [20,22,23].

Several studies have reported that the application of Ca^2+^ inhibits the K^+^ absorption or transportation in plants [24–26]. However, inhibitory effects of Ca^2+^ on K^+^ absorption are temporary and time-dependent because Ca^2+^ is needed to maintain the integrity of cellular membranes and of selective ion transport mechanisms by which K^+^ is actively absorbed [17,21,25,27,28]. Research has also shown that Ca^2+^ can significantly promote the absorption of K^+^ [25]. Moreover, some research has been done on the interaction between nutrients [29–31] and also on the influence of nutrients under heavy metals stress [32–35]. However, the mechanism involved in absorption and transport of heavy metals in plants is still unclear related to the soil environment [10]. Therefore, it is important to study the interactions between nutrients and heavy metals. However, scanty information is available on the efficacy of Ca^2+^ and K^+^ in offsetting the Cd stress in plants. Thus, we focused on Ca^2+^ and K^+^ as possible inducers in the tolerance of faba bean to Cd stress. The major objectives of our study were to determine the interactive efficiency of Ca^2+^ and K^+^ treatments in restoring the metabolic alterations resulting from Cd stress in faba bean.

## 2. Results

**Figure 1:**
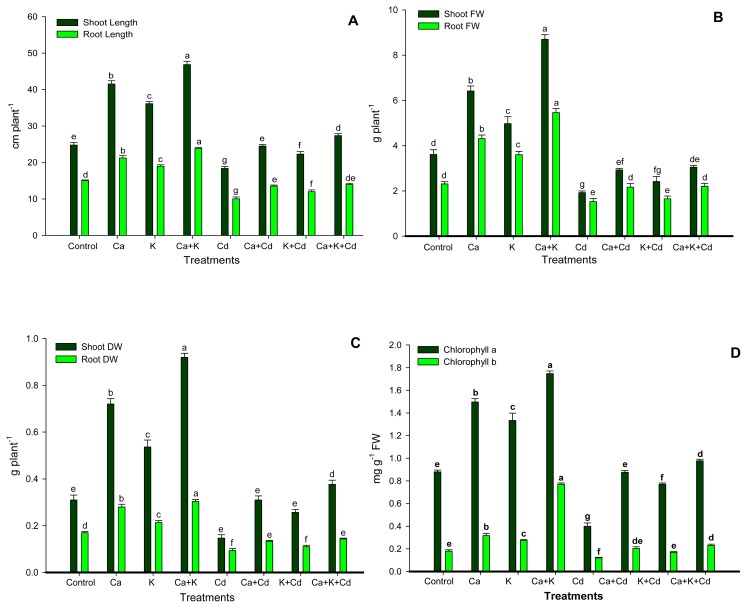
Ameliorating effect of calcium and potassium on shoot and root length (**A**), shoot fresh weight (FW) and root FW (**B**), shoot dry weight (DW) and root DW (**C**) and Chl *a* and Chl *b* (**D**) of faba bean plants under Cd stress. Bars followed by the same letters show no statistical difference at *p* < 0.05 (Duncan Multiple Range Test). Average of four determinations are presented with bars indicating SE.

The significant changes of growth parameters in faba bean plants subjected to Ca^2+^ and/or K^+^ under Cd stress and normal conditions are shown in Figure 1A–C. Application of Ca^2+^ and K^+^ individually as well as in combination improved root length (RL) plant^−1^, shoot length (SL) plant^−1^, root fresh weight (RFW) plant^−1^, shoot fresh weight (SFW) plant^−1^, root dry weight (RDW) plant^−1^ and shoot dry weight (SDW) plant^−1^], as compared with the controls. However, combined application of Ca^2+^ and K^+^ showed more enhancing effect on all growth traits than individual treatment. Application of Cd inhibited all growth attributes of faba bean. However, the subsequent treatment of Cd-stressed plants with Ca^2+^ or K^+^ alone as well as in combination alleviated the adverse effect of Cd on faba bean and caused a considerable improvement of RL, SL, SFW, RFW, SDW, and RDW. The combined application of Ca^2+^ and K^+^ had maximum alleviating effect of Cd toxicity on all growth characteristics when compared with Ca^2+^ and K^+^ supplied alone. In contrast, combined application of Ca^2+^ and K^+^ showed parity with the application of Ca^2+^ alone for SFW and RFW.

Figure 1D reveals that application of Ca^2+^ and K^+^ alone as well as together significantly increased the chlorophyll (Chl) *a* and Chl *b* as compared to the control under normal conditions. The combined application of Ca^2+^ and K^+^ exhibited maximum value for both Chl *a* and Chl *b* when compared with individual application of Ca^2+^ and K^+^, under normal conditions. However, presence of Cd in growth medium suppressed both the photosynthetic pigments. Application of Ca^2+^ and/or K^+^ significantly enhanced Chl *a* and Chl *b* under stress. Moreover, application of Ca^2+^ and K^+^ together was found to be more effective in alleviating the adverse effect of Cd stress on Chl *a* and Chl *b*.

**Figure 2:**
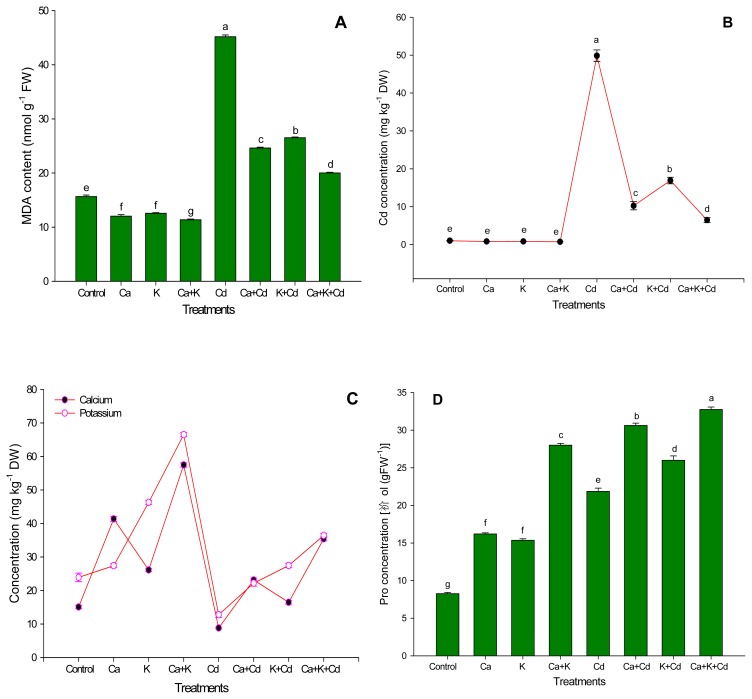
Ameliorating effect of calcium and potassium on MDA content (**A**), Cd content (**B**), Ca^2+^ and K^+^ content (**C**) and Pro content (**D**) of faba bean plants under Cd stress. Bars followed by the same letters show no statistical difference at *p < 0.05* (Duncan Multiple Range Test). Average of four determinations are presented with bars indicating SE.

Figure 2A showed that Cd supplied in growth medium triggered accumulation of MDA, when compared with control. In contrast, under normal conditions, application of Ca^2+^ and/or K^+^ inhibited accumulation as compared to the control. However, application of Ca^2+^ and K^+^ alone as well as in combination arrested the ill effect of Cd up to a considerable limit by decreasing the accumulation of MDA in the leaf of faba bean plants. Under stress, the lowest accumulation of MDA was recorded in the plants that received Ca^2+^ and K^+^ together.

Application of Cd increased the highest content of Cd in leaves as compared with control and other treatments (Figure 2B). However, under Cd stress, application of Ca^2+^ and K^+^ alone was found to be effective in suppressing leaf-Cd concentration, but maximum inhibition of Cd content was recorded in plants fed with the combined Ca^2+^ and K^+^ application. The presence of Cd in growth medium suppressed both the content of both nutrients in the leaf. However, application of Ca^2+^ and K^+^ alone as well as in combination increased leaf-Ca^2+^ and K^+^ content under normal as well as stress conditions. Application of Ca^2+^ and K^+^ individually was found to be effective in improving the content of leaf-Ca^2+^ and K^+^, but degree of efficiency of combined application of Ca^2+^ and K^+^ in alleviating the adverse effect of Cd stress on leaf-Ca^2+^ and K^+^ content was found to be maximum (Figure 2C).

It is evident from Figure 2D that proline (Pro) was higher in plant treated with combined application of Ca^2+^ and K^+^. Application of Ca^2+^ and K^+^ individually on unstressed plants could not bring about a significant change from control in the level of Pro. However, in association with Cd, they improved the quantity of Pro. Under stress conditions, application of Ca^2+^ gave maximum value for Pro when compared with K^+^ applied alone. The highest level of Pro was found in stressed plants, which were subjected to Ca^2+^ and K^+^ together.

**Figure 3:**
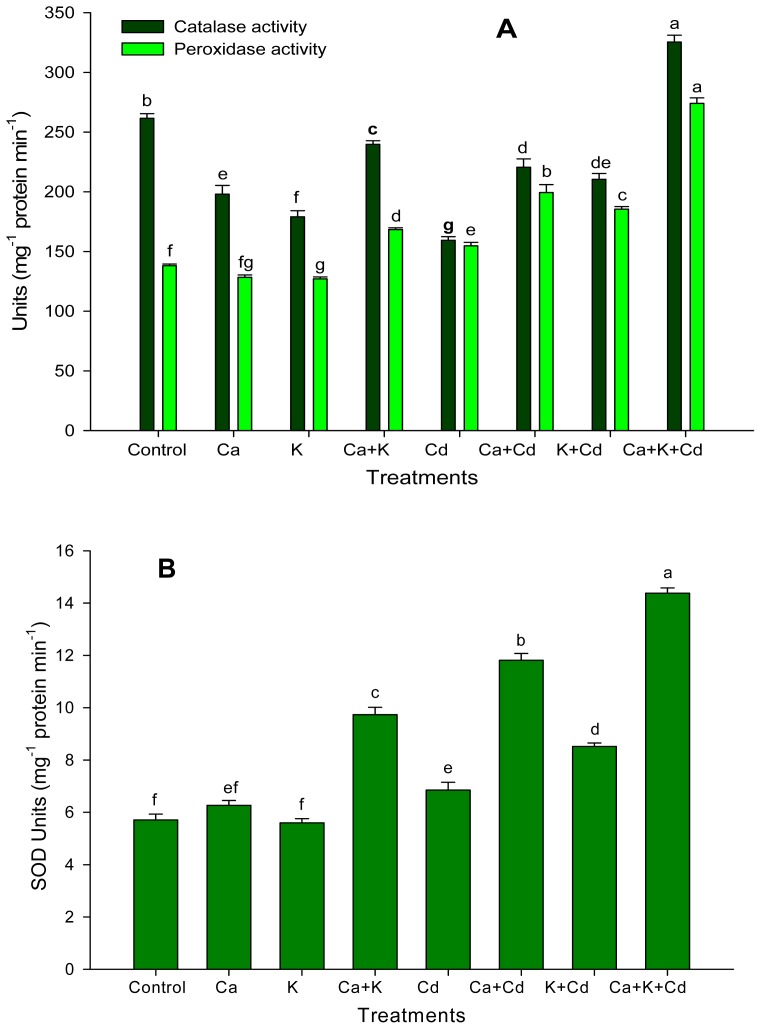
Ameliorating effect of calcium and potassium on the activity of (**A**) antioxidant enzymes catalase (CAT) and peroxidase (POD) and (**B**) superoxide dismutase (SOD) of faba bean plants under Cd stress. Bars followed by the same letters show no statistical difference at *P<0.05* (Duncan Multiple Range Test). Average of four determinations are presented with bars indicating SE.

The changes in the activity of antioxidant enzymes, *i.e.*, catalase (CAT), peroxidase (POD), and superoxide (SOD) in faba bean plants exposed to Ca^2+^ and/or K^+^ under both stress and non-stress conditions are shown in Figures 3A and 3B. Under non-stress conditions, application of Ca^2+^ and K^+^ individually decreased the activity of CAT and POD except SOD, but they gave a maximum value for these enzymes’ activity when they were applied together. Under Cd stress, application of Ca^2+^ individually gave maximum value for POD and SOD, but its effect was at par with K^+^ alone for CAT activity. However, the subsequent application of Cd-stressed plants with the combination of Ca^2+^ and K^+^ neutralized the adverse effect of Cd and resulted in considerable improvement in the activities of these three enzymes compared to their individual treatment (Figure 3).

## 3. Discussion

It is well established that accumulation of Cd in plant tissues may cause a changes in various physiological processes resulting in poor growth, development and productivity of plants [1,9,36]. In the present experiment, plants subjected to Cd showed reduced growth in terms of RL, SL, RFW, SFW, RDW and SDW (Figures 1A–1C). The inhibition of growth traits may be due to the consequence of alteration of photosynthesis rate, and uptake and distribution of essential nutrients [37–39]. In the present study, application of Ca^2+^ and K^+^ alone as well as in combination was efficient to restore the altered plant growth induced by Cd toxicity in *Vicia faba*. The ameliorating response of both nutrients on plant growth is the consequence of cell elongation and cell division [21,40]; K^+^ acts as catalyst, even under adverse conditions, for many of the enzymatic processes that are necessary for plant growth and development [41]. Khan *et al.* [11] andSiddiqui *et al.* [35] reported that presence of Ca^2+^ in nutrient medium containing NaCl and Ni, promoted the plant growth. Thus, on the basis of the roles played by these nutrients, we could easily visualize their direct or indirect role in plant growth. This, in turn, could be responsible for the reversal of the altered plant growth induced by Cd stress; these improvements lead the plants with better adaptation under different adverse conditions. Thus, we may postulate that Ca^2+^ and K^+^ applied together in the present study were more effective in the restoration of altered plant growth characteristics (Figure 1).

The photosynthetic pigments are important macromolecules that are produced by plants, and play a vital role in photosynthesis, which is responsible for plant growth and dry matter production. In the present study, reduction of Chl *a* and *b* concentration induced by Cd stress might be due to the instability of proteins complex, destruction of chloroplast and photosynthetic apparatus [42], inhibition of photosynthetic electron transport chain [43] and the enzymes of Chl biosynthesis such as δ-aminolevulinic acid (ALA) synthase, ALA dehydratase, and porphobilinogenase [44]. Cadmium is involved in the inhibition of heme biosynthesis and chlorophyll synthesis by interacting with sulphydryl requiring enzymes involved in the pathway [45]. Interestingly, from this finding, it is very clear that the application of Ca^2+^ and/or K^+^ significantly enhance the concentration of these pigments under stress and non-stress conditions. Photosynthetic pigments could be increased due to the maximum presence of leaf-Ca^2+^ (Figure 2B) that could act as a secondary messenger for cytokinin action in promoting the Chl biosynthesis; Ca^2+^ could interact with light in the pathway of Chl synthesis [46]. Also, K^+^ plays an important role in the formation of photosynthetic pigment by preventing decomposition of newly formed Chl and ALA formation [47]. This result indicates that combined application of Ca^2+^ and K^+^ is associated with tolerance of faba bean plants to Cd stress by improving the biosynthesis of photosynthetic pigments.

In the present study, an elevated level of MDA concentration in leaves indicates that the plants were subjected to Cd stress induced lipid peroxidation, which resulted in membrane damage (Figure 2A). This is in accordance with other findings [48,49]. However, the plants that were subjected to Ca^2+^ and K^+^ alone as well as in combination, exhibited lowest values for MDA content (Figure 2A). This may be due to the antioxidant enzymes activity and Pro accumulation (Figures 2D and 3A, 3B) which has an adaptive significance, as they detoxify the oxygen free radicals and thus reduce the oxidative stress linked membrane deterioration under Cd stress. Also, the inhibition of MDA content may be due to the role of Ca^2+^ in controlling membrane structure and function by binding to phospholipids that stabilizes lipid bilayers and thus provides structural integrity to cellular membranes [21]. Furthermore, there is the possibility of the involvement of oxidative signal transduction concomitant with the regulation of antioxidant enzymes under stress [17,50]. Also, K^+^ plays a key role in reduction of ROS production by reducing activity of NAD(P)H oxidases and maintaining photosynthetic electron transport [41].

The accumulation of Cd in leaves of faba bean was high under Cd stress (Figure 2B). However, the accumulation of Cd was found to be lower in plants subjected to Ca^2+^ and K^+^ alone as well as in combination (Figure 2C). In this study, the highest accumulation of Ca^2+^ and K^+^ in leaf was recorded with the combined application of Ca^2+^ and K^+^, under stress and non-stress conditions. As evidenced in the present study, Cd caused a decrease in leaf-Ca^2+^ and K^+^ content in the plants (Figure 2C) which accords with the findings of Suzuki [15] and Umar *et al.* [51]. In Ca^2+^plus K^+^ treated plants, the increased accumulation of Ca^2+^ and K^+^ may be responsible for the inhibition of uptake and deleterious effects of Cd in Faba bean plants. The improvement of tolerance of plant to Cd stress may be due to the presence of Ca^2+^ that is responsible for active exclusion of toxic Cd by the formation and excretion of Cd/Ca^2+^ containing crystals through the head cells of trichomes [52]. It was also previously reported that Ca^2+^ was able to alleviate Cd toxicity, presumably through competition for metal ion influx [15]. Under abiotic stress, plants also require more K^+^ to maintain the photosynthesis because, during this process, reactive oxygen species (ROS) is generated [41]. The increased accumulation of Ca^2+^ and K^+^ with the application of Ca^2+^ and K^+^ alone as well as in combination have been major factors for increasing DW production and plant height because both are important components of many metabolically important compounds and play a vital role in various physiological processes [12].

Pro is not only a universal osmoprotectant, but also acts as an antioxidant as well as a source of energy; also it is considered to be an important biomolecule that has a protective role in tolerance of plant to abiotic stresses [16,35,53,54]. Cd stress leads to protein degradation through amino acid metabolism resulting in decreased plant growth [55]. In the present study, under Cd stress, accumulation of Pro was increased as compared to the control (Figure 2D) which is in accordance with the finding of Zhao [54]. However, application of Ca^2+^ and K^+^ individually as well as in combination was found to be effective to enhance further accumulation of Pro under stress and non-stress conditions (Figure 2D). The enhanced accumulation of Pro in leaves may represent a major biochemical adaptation, membrane stabilization and ROS scavenger [16,30,35,53,56]. Free Pro acts as an antioxidant in Cd-stressed cells and Pro levels are correlated with the GSH redox state and MDA levels in heavy metal-treated algae [57]. This result agrees with the finding of Siddiqui *et al.* [16], who reported that the production of Pro was higher in Ca^2+^-exposed plants under heavy metal stress.

The antioxidant system is one of the important defense mechanisms of plants, through which plants perform normally under different environmental conditions by scavenging ROS. In this experiment, antioxidant enzymes such as POD and SOD except CAT, increased slightly under Cd stress (Figures 3A, 3B). However, application of Ca^2+^ and K^+^ significantly enhanced the activity of all enzymes; the fact that the maximum increase in the activity of these enzymes was found in plants might be due to the combined application of Ca^2+^ and K^+^ (Figures 2A, 2B). The increased activity of antioxidant enzymes may be due to the active role of K^+^, which activates more than 50 enzymes [14]. Under stress, K^+^ plays important role in the synthesis of protein by participating in polypeptide synthesis in the ribosomes, since that process requires a high concentration of K^+^ [58]. Tripathi *et al.* [59] reported that proteins, such as thioredoxin, glutaredoxin, cyclophilin, among others, are known to facilitate the regeneration of the reduced (catalytically active) form of peroxiredoxins that play an important role in reducing the ROS formation in plants under biotic and abiotic stress. Also, Ca^2+^ acts as a secondary messenger of external stimuli, and stimulates calmodulin-like proteins that interact with Ca^2+^ ions. Changing their conformation in response to Ca^2+^ binding, calmodulin proteins regulate a variety of mechanisms, including ion transport, gene regulation, cell motility, growth, proliferation, apoptosis and stress tolerance that coordinate, at least in part, the plant response to Cd [60–62]. The Ca^2+^ might be responsible for decreased thiobarbutric acid and hydrogen peroxide content, as the unique importance of Ca^2+^ for stabilizing membranes is well established [21]. Khan *et al.* [17] and Siddiqui *et al.* [16] reported that application of Ca^+2^ was found to be effective in improving the tolerance of plant to abiotic stress by improving antioxidant systems. Thus, these results indicate that the improved activity of antioxidative enzymes due to the combined application of Ca^2+^ and K^+^ resulted in an increase in the capacity of various defense mechanisms and also improvement in various physiological and biochemical processes leading to the tolerance of the plant to Cd stress.

## 4. Experimental Section

### 4.1. Plant Cultures and Treatments

To meet the objectives mentioned in the introduction, the response of fabe bean (*Vicia faba* L.*)* to Ca^2+^ and K^+^ under Cd toxicity were studied by conducting a greenhouse pot experiment at the Department of Botany and Microbiology, King Saud University, Riyadh. Seeds of faba bean (cv. TARA) were obtained from a local market in Riyadh, Saudi Arabia. Healthy seeds were surface sterilized by using 1% sodium hypochlorite for 10 min then vigorously rinsed with sterilized double distilled water (DDW) before sowing. The seeds were sown in plastic pots (25 cm in diameter, 25 cm height) filled with perlite and supplied with Raukura’s nutrient solution [63]. The pots were arranged in a simple randomized design in the Glasshouse (Department of Botany and Microbiology, King Saud University, Riyadh, KSA) with a single factor and four replicates. The pots were covered with black plastic to reduce evaporation. One week after sowing, seedlings were thinned so that each pot contained healthy plants of uniform size. Pots were irrigated every two days with DDW (100 mL) to keep the perlite moist. Ca and K treatments were initiated 10 days after germination as follows: (i) Ca_0_+K_0_+Cd_0_ (control); (ii) Ca_40_ mM + K_0_ + Cd_0_; (iii) Ca_0_ + K_6_ mM + Cd_0_; (iv) Ca_40_ mM + K_6_ mM + Cd_0_; (v) Ca_0_ + K_0_ +Cd_200_ μM; (vi) Ca_40_ mM + K_0_ + Cd_200_ μM; (vii) Ca_0_ + K_6_ mM + Cd_200_ μM; (viii) Ca_40_ mM + K_6_ mM + Cd_200_ μM. The sources of Ca and K were calcium chloride and potassium chloride, respectively. Plants were sampled on the seventh day after treatment to assess their growth characteristics SL plant^−1^, RL plant^−1^, SFW plant^−1^, and RFW plant^−1^, SDW plant^−1^, RDW plant^−1^ and physiological attributes [Chl *a* and Chl *b*, concentrations of Ca^2+^, K^+^, Pro and MDA; and activity of CAT, POD and SOD].

### 4.2. Plant Growth Characteristics

Shoot length and RL were measured using a meter scale after removal from the pots. After recording FW with balance, plants were placed in a 60 °C oven for 48 h and then were weighed for DW.

### 4.3. Physiological and Biochemical Parameters

#### 4.3.1. Chemical Content of Leaves

All the chemical reagents used in this procedure were of analytical grade. Absorbances were determined using a UV-VIS spectrophotometer, unless otherwise specified. Chlorophyll was extracted from fresh leaves using the DMSO method of Barnes *et al.* [64]. Chl *a* and Chl *b* concentrations were calculated based on the absorbance of the extract at 663.8 and 646.8 nm.

To determine Ca, K and Cd concentrations, we followed the digestion approach of Zheljazkov and Nielson [65] as modified by Hseu [66]. A leaf sample (0.5 g) was placed in a 250 mL digestion tube, and 10 mL of 2:1 concentrated nitric acid: perchloric acid was added. Samples were heated for 45 min at 90 °C for 2–3 h until a clear solution was obtained. At intervals, 5 mL of concentrated nitric acid: perchloric acid with hydrogen peroxide were added to the sample (at least three times), and the digestion continued until the volume was reduced to about 1 mL. The interior walls of the tube were washed down with a little DDW and the tubes were swirled throughout the digestion to keep the walls clean and prevent loss of the samples. After cooling, 5 mL of 1% HNO_3_ was added to each sample. Thereafter, the solution was filtered through Whatman No. 42 filter paper and <0.45 μm millipore filter paper. The filtrate was diluted to a total of 25 mL with distilled water. After dilution, the concentrations of Ca and K were determined by using with an atomic absorption spectrometer (Model 300, Perkin-Elmer, Waltham, MA, USA) and Cd was determined with the help of Inductively Coupled Plasma Optical Emission Spectroscope (Model iCAP6000, Thermo-Scientific, Thermo-Fisher Scientific, Waltham, MA, USA).

Proline concentration was determined spectrophotometrically by adopting the ninhydrin method of Bates *et al.* [67]. We first homogenized 300 mg of fresh leaf samples in sulphosalicylic acid, then added 2 mL each of acid ninhydrin and glacial acetic acid. The samples were heated at 100 °C. The mixture was extracted with toluene and the free toluene was quantified at 528 nm using L-proline as a standard.

Malondialdehyde (MDA) content was determined according to the method of Heath and Packer [68]. Leaves were weighed and homogenates containing 10% trichloroacetic acid (TCA) and 0.65% 2-thiobarbituric acid were heated at 95 °C for 60 min then cooled to room temperature and centrifuged at 10,000 × *g* for 10 min. The absorbance of the supernatant was read at 532 nm and 600 nm against a reagent blank.

#### 4.3.2. Enzyme Activity

To determine the enzymatic activities of the antioxidant proteins, a crude enzyme extract was prepared by homogenizing 500 mg of leaf tissue in extraction buffer containing 0.5% Triton X-100 and 1% polyvinylpyrrolidone in 100 mM potassium phosphate buffer (pH 7.0) using a chilled mortar and pestle. The homogenate was centrifuged at 15,000 × *g* for 20 min at 4 °C. The supernatant was used for the enzymatic assays described below. All enzyme activities were expressed as units mg^−1^ protein min^−1^.

We used the method of Chance and Maehly [69] to determine POD (E.C. 1.11.1.7) activity by using 5 mL of an assay mixture containing phosphate buffer (pH 6.8), 50 M of pyrogallol, 50 mM of H_2_O_2_, and 1 mL of the enzyme extract diluted 20×. This was incubated for 5 min at 25 °C, after which the reaction was stopped by adding 0.5 mL of 5% (v/v) H_2_SO_4_. The amount of purpurogallin formed was determined by measuring absorbance at 420 nm. A unit of peroxidase activity was the amount of purpurogallin formed per mg protein per minute.

Aebi [70] method was used to measure CAT (EC 1.11.1.6) activity. The decomposition of H_2_O_2_ was monitored by the decrease in absorbance at 240 nm. For the assay, a 50 mM phosphate buffer (pH 7.8) and 10 mM H_2_O_2_ were used.

The activity of SOD (EC 1.15.1.1) was determined by measuring its ability to inhibit the photoreduction of nitro blue tetrazolium (NBT) according to the methods of Giannopolitis and Ries [71]. The reaction solution (3 mL) contained 50 μmol NBT, 1.3 μmol riboflavin, 13 mmol methionine, 75 nmol EDTA, 50 mmol phosphate buffer (pH 7.8), and 20 to 50 μL enzyme extract. The reaction solution was irradiated under a bank of fluorescent lights at 75 μmol·m^−2^·s^−1^ for 15 min. The absorbance at 560 nm was read against a blank (non-irradiated reaction solution). One unit of SOD activity was defined as the amount of enzyme that inhibited 50% of NBT photoreduction.

### 4.4. Statistical Analysis

Each pot was treated as one replicate and all the treatments were repeated four times. The data were analyzed statistically with SPSS-17 statistical software (SPSS Inc., Chicago, IL, USA). Means were statistically compared by Duncan’s Multiple Range Test (DMRT) at the *p* < 0.05% level.

## 5. Conclusions

In summary, results presented above confirm the importance of Ca^2+^ and/or K application in the detoxification of Cd toxicity. These results clearly show that application of Ca^2+^ and/or K^+^ decreased in MDA and Cd contents, and increased in photosynthetic pigment, Pro, antioxidant enzymes activity (CAT, POD and SOD) and accumulation of nutrients (Ca^2+^ and K^+^) may be responsible for better growth in terms of RL, SL, RFW, SFW that were responsible for enhanced dry matter production under Cd stress. Therefore, inclusion of Ca^2+^ and/or K^+^ in growth medium could be an adequate strategy to alleviate the harmful effects of heavy metal stress and to enhance plant metabolism to perform better under normal as well as different environmental conditions.
